# Stereoselective block of the hERG potassium channel by the Class Ia antiarrhythmic drug disopyramide

**DOI:** 10.1007/s00018-024-05498-4

**Published:** 2024-11-28

**Authors:** Yihong Zhang, Aziza El Harchi, Andrew F. James, Shigetoshi Oiki, Christopher E. Dempsey, Jules C. Hancox

**Affiliations:** 1 School of Physiology, Pharmacology and Neuroscience, Biomedical Sciences Building, University of Bristol, University Walk, Bristol, BS8 1TD UK; 2 Biomedical Imaging Research Centre, University of Fukui, 23-3 Matsuokashimoaizuki, Eiheiji-cho, Fukui, 910-1193 Japan; 3 School of Biochemistry, Biomedical Sciences Building, University of Bristol, University Walk, Bristol, BS8 1TD UK

**Keywords:** Disopyramide, Enantiomer, HERG, Long QT, QT interval, Stereoselectivity

## Abstract

Potassium channels encoded by *human Ether-à-go-go-Related Gene* (*hERG*) are inhibited by diverse cardiac and non-cardiac drugs. Disopyramide is a chiral Class Ia antiarrhythmic that inhibits hERG at clinical concentrations. This study evaluated effects of disopyramide enantiomers on hERG current (I_hERG_) from hERG expressing HEK 293 cells at 37 °C. S(+) and R(−) disopyramide inhibited wild-type (WT) I_hERG_ with IC_50_ values of 3.9 µM and 12.9 µM respectively. The attenuated-inactivation mutant N588K had little effect on the action of S(+) disopyramide but the IC_50_ for the R(−) enantiomer was ~ 15-fold that for S(+) disopyramide. The enhanced inactivation mutant N588E only slightly increased the potency of R(−) disopyramide. S6 mutation Y652A reduced S(+) disopyramide potency more than that of R(−) disopyramide (respective IC_50_ values ~ 49-fold and 11-fold their WT controls). The F656A mutation also exerted a stronger effect on S(+) than R(−) disopyramide, albeit with less IC_50_ elevation. A WT-Y652A tandem dimer exhibited a sensitivity to the enantiomers that was intermediate between that of WT and Y652A, suggesting Y652 groups on adjacent subunits contribute to the binding. Moving the Y (normally at site 652) one residue in the N- terminal (up) direction in N588K hERG markedly increased the blocking potency of R(−) disopyramide. Molecular dynamics simulations using a hERG pore model produced different binding modes for S(+) and R(−) disopyramide consistent with the experimental observations. In conclusion, S(+) disopyramide interacts more strongly with S6 aromatic binding residues on hERG than does R(−) disopyramide, whilst optimal binding of the latter is more reliant on intact inactivation.

**Supplementary Information:**

The online version contains supplementary material available at 10.1007/s00018-024-05498-4.

## Introduction

Potassium channels encoded by the *human Ether-à-go-go Related Gene (hERG*; alternative nomenclature *KCNH2*) carry the rapid delayed rectifier potassium current, I_Kr_, that controls repolarization of ventricular action potentials (APs) in the heart [1–3]. Loss-of-function *hERG* mutations give rise to the LQT2 form of congenital long QT syndrome (LQTS), whilst gain-of-function mutations underpin the SQT1 form of short QT syndrome (SQTS) [1, 3]. hERG/I_Kr_ channels are also a target both for antiarrhythmic (Class Ia and III) drugs and numerous drugs associated with drug-induced LQTS and the associated ventricular arrhythmia *Torsades de Pointes* (TdP) [1, 3]. This association is sufficiently strong that all novel pharmaceuticals must be tested for an ability to inhibit hERG channel ionic current, I_hERG_ [1, 3]. The hERG channel’s remarkable sensitivity to pharmacological inhibition has been linked to interactions with aromatic residues (Y652 and F656) in the channel’s S6 helices and residues near the base of the selectivity filter [1, 4, 5], with recent work also demonstrating the presence of hydrophobic pockets below the selectivity filter that may also form drug-interaction sites [6, 7]. Intact inactivation gating is important for I_hERG_ inhibition by a number of high affinity blockers, although some other drugs have a comparatively low dependence on intact inactivation for inhibition of I_hERG_ to occur (e.g. [8, 9]).

Disopyramide (4-(diisopropylamino)−2-phenyl-2-(pyridin-2-yl) butanamide) is a Class Ia antiarrhythmic drug that has long been used in the treatment of both supraventricular and ventricular arrhythmias (e.g.[10–14]). It is as effective as propafenone at maintaining sinus rhythm after cardioversion of chronic atrial fibrillation (PRODIS; [15]) and is similarly effective to mexiletine in reducing frequency of ventricular premature contractions [11]. Disopyramide can also be valuable in the treatment of hypertrophic cardiomyopathy [16–18]. The drug’s use is associated with a modest, albeit significant risk of TdP, associated with QT interval prolongation [19–22]. The ability of disopyramide to delay ventricular repolarization and prolong the QT interval is strongly linked to its propensity to inhibit native I_Kr_ and underlying hERG channels at clinically relevant concentrations [23, 24]. However, disopyramide differs from canonical high affinity hERG channel inhibitors in that its I_hERG_ inhibitory action has a comparatively weak dependence on channel inactivation [9, 24]. Due to this, the drug has been found to be an effective inhibitor of attenuated-inactivation, gain-of-function hERG channel mutants that cause SQTS [9, 25, 26]. Data from mutagenesis experiments indicate a strong contingency of I_hERG_ inhibition by disopyramide on drug interactions with S6 aromatic residues (F656 and Y652), but without concomitant interactions with residues at the base of the pore helix found to be important for actions of high affinity I_hERG_ inhibitors [27].

Disopyramide is chiral and there is some evidence that the two enantiomers may differ in respect of their effects on cardiac repolarization. For example, in rabbits, the S(+) enantiomer of disopyramide has been reported to be ~ threefold more potent than the R(−) enantiomer in prolonging the QU_c_ interval [28]. The acute infusion of the S(+) but not R(−) enantiomer into healthy human volunteers has been reported to result in prolongation of the rate corrected QT interval, [29]. It is notable that hERG channel inhibition by the local anaesthetic bupivacaine and the synthetic opiate methadone have been reported to exert stereoselective hERG inhibition [30–33]. However, in contrast there is a lack of similar information for disopyramide. It has been noted by others that investigation of whether disopyramide inhibition of hERG exhibits stereoselectivity might lead to reconsideration of the safety of administration of the racemate [32]. Furthermore, the dependence of disopyramide block on S6 aromatic residue interactions without accompanying interactions with residues below the pore helix makes disopyramide an attractive molecule with which to investigate stereoselectivity of drug interactions with the Y652 and F656 residues. Accordingly, this study was undertaken to compare I_hERG_ inhibitory potency of the S(+) and R(−) disopyramide enantiomers and has identified the S(+) enantiomer to be the more potent I_hERG_ inhibitor of the two. Marked differences were found in effects of mutations that help explain the differential effects of the two disopyramide enantiomers.

## Materials and methods

### Molecular biology and mutagenesis

Construction and use of the hERG tandem dimer construct (D-Y652A) has been described previously [34, 35]. Briefly, this construct was produced by linking N-terminal side wild-type (WT) monomer and C-terminal side S6 mutant Y652A monomer by the nucleotide sequence GAATTC (see Fig. 4A) [34, 35].

The WT and N588K hERG constructs used in this study have been described previously [25, 36]. The N588E hERG mutant [8] was engineered in modified pcDNA3.0 expression vector (kindly donated by Dr John Mitcheson), using QuikChange^®^ (Stratagene) mutagenesis. The following primer sequences were used:

forward primer 5’CGG CTG GCT GCA CGA GCT GGG CGA CCA GA3’

reverse primer 5’TCT GGT CGC CCA GCT CGT GCA GCC AGC CG3’

we used our existing hERG N588K mutant in our modified pCDNA3.0 vector [36] as template to generate mutants with reposition of aromatic Tyr(Y) or Phe(F) residues in the S6 helix by one position, either toward the N or C terminus as shown in Fig. 5A, in each case an aromatic residue (normally positioned at Y652 or F656 of the wild-type channel) was replaced with an Ala(A) and a neighbouring residue (e.g., Ile-655) mutated to the aromatic residue. The resulting mutant channel was named according to whether the aromatic residue was transferred in the N-terminal (“up”) or C-terminal (“down”) direction (cf [37]). N588K-Y-up, -Y-down, -F-up and -F-down mutated channels were constructed using QuikChange® (Stratagene) mutagenesis as previously reported [38, 39].

The following forward primer sequences were used:

5’CTCATTGGCTCCCTCTATGCTGCTAGCATCTTCGGC-3’for N588K-Y-up, 5’GGCTCCCTCATGGCTTATAGCATCTTCGGC-3’ for N588K-Y-down,

5’-CTCATGTATGCTAGCTTCGCCGGCAACGTGTCGG-3’for N588K-F-up

5’CTCATGTATGCTAGCATCGCCTTCAACGTGTCGGCCATCATC-3’for N588K-F-down.

In each case, competent DH5α Escherichia coli (Invitrogen, Paisley, UK) was transformed using standard procedures, DNA was purified using Endotoxin‐free plasmid DNA purification kit (Neumann‐Neander‐Str., Germany, Macherey‐Nagel), DNA was sequenced for the full length of the hERG insert to ensure that only the correct mutation had been made (Eurofins WG Operon, Ebersberg, Germany).

### Maintenance of cells and cell transfection

Human embryonic kidney (HEK 293) cells stably expressing WT hERG (generously donated by Dr Craig January, University of Wisconsin; [40]) or hERG mutants F656A and Y652A [39, 41] were employed. Stable cell lines were cultured with a modification of Dulbecco minimum essential medium with Glutamax-1 (DMEM; Gibco, Paisley, UK). This was supplemented with 10% fetal bovine serum, 50 μg/mL gentamycin (Invitrogen, Paisley, UK), and 400 μg/mL geneticin (G418, Invitrogen, Paisley, UK) for WT or 100 μg/mL of hygromycin for Y652A and F656A, cells were passaged using enzyme-free cell dissociation solution (Millipore, Watford, UK) and plated onto sterilized 13-mm glass coverslips in 40 mm Petri dishes. The cells were incubated at 37 °C (5% CO_2_) for a minimum of 1 day before any electrophysiological study [27, 38, 39].

For other mutants, HEK 293 cells (ECACC, Porton Down, UK) were transiently transfected with 1 μg of hERG cDNA using Lipofectamine 2000 (Invitrogen, Paisley, UK) according to the manufacturer’s instructions. Expression plasmid encoding CD8 (0.15 μg) was also added (in pIRES, donated by Dr I Baró, University of Nantes, France) to be used as a successful marker of transfection. Successfully transfected cells (positive to CD8) were identified using Dynabeads^®^ (Invitrogen, Paisley, UK). Electrophysiological recording experiments were performed 24–48 h after transfection [27, 38, 39].

### Electrophysiological recording

Whole‐cell recordings were made at 37 ± 1 °C by using an Axopatch 200B amplifier (Axon Instruments, Foster City, CA, USA). Patch pipettes were fire polished to 2.5–4 MΩ. Between 70 and 80% of the electrode series resistance could be compensated. Data were recorded via a Digidata 1440A interface (Molecular Devices, Sunnyvale, CA, USA). Once in the chamber, cells were continuously superfused with an external solution containing (in mM): 140 NaCl, 4 KCl, 2.5 CaCl_2_, 1 MgCl_2_, 10 Glucose and 5 HEPES (titrated to pH 7.45 with NaOH). For experiments employing the S6 mutants F656A and their WT control, the external solution contained 94 mM KCl (the NaCl concentration was correspondingly reduced). The pipette dialysate contained (in mM): 130 KCl, 1 MgCl_2_, 5 EGTA, 5 MgATP, 10 HEPES (titrated to pH 7.2 using KOH) [26, 27, 38, 39].

### Disopyramide enantiomers

Disopyramide enantiomers (S(+) disopyramide and R(−) disopyramide) were manufactured by Custom Synthesis, Abcam (formerly Ascent Scientific Ltd, Bristol, UK), by resolution from the racemate. Chiral separation was performed by high-performance liquid chromatography from racemic disopyramide-phosphate (Sigma, UK) and for each enantiomer peak purity was > 99%. Enantiomers were recrystallised to constant rotation as the bitartrate salt. S(+) disopyramide and R(−) disopyramide were dissolved in methanol to produce stock solution of 100 mM which was then diluted to produce stock solutions ranging down to 1 mM. The disopyramide stock solutions were diluted at least 1:1000-fold with Tyrode’s solution to achieve concentrations stated in the ‘Results’. External solutions were applied using a home-built, warmed and rapid solution exchange device [42].

### Electrophysiology data analysis

Data were analyzed using Clampfit 10.2 (Axon Instruments) or WinWCP v5.2 (Strathclyde Electrophysiology Software), Excel 2016 (Microsoft, Redmond, WA), Origin 2018b (OriginLab Corporation, Northampton, MA, USA), Prism 5 and Prism 8 (Graphpad Inc, La Jolla, CA, USA) and Corel Draw (CorelDraw Graphics) software. Data are presented as the mean ± standard error of the mean (SEM). Statistical comparisons were made using the Student’s *t*-test, or two-way analysis of variance (ANOVA) followed by Bonferroni post hoc test, as appropriate. *p* values less than 0.05 were taken as being statistically significant.

The equations described below were used for numerical analysis and graphical fits to data sets [38, 39] [43]:

1 $${\text{Fractional block}}\, = \,{1}\, - \,\left( {{\text{I}}_{{{\text{hERG}} - {\text{drug}}}} /{\text{I}}_{{{\text{hERG}} - {\text{control}}}} } \right)$$

where Fractional block refers to the degree of inhibition of hERG current by a given concentration of drug. I_hERG-drug_ and I_hERG-control_ represent “tail” current amplitudes in the presence and absence of drug.

Concentration–response data were fitted by a standard Hill equation of the form:

2 $${\text{Fractional block}}\, = \,{1}/\left( {{1}\, + \,\left( {{\text{IC}}_{{{5}0}} /\left[ {{\text{drug}}} \right]} \right)^{h} } \right)$$

where IC_50_ is [drug] producing half-maximal inhibition of the I_hERG_ tail and *h* is the Hill coefficient for the fit.

Half‐maximal activation voltage values were obtained by normalizing I_hERG_ tail values (I) at − 40 mV following differing voltage commands to the maximal I_hERG_ tail value observed during the voltage protocol (Imax). The resulting values were plotted against corresponding command voltage (Vm), and fitted by a Boltzmann equation of the form:

3 $${\text{I}}/{\text{Imax}}\, = \,{1}/\left( {{1}\, + \,{\text{exp}}\left( {\left( {{\text{V}}_{{0.{5}}} - {\text{Vm}}} \right)/{\text{k}}} \right)} \right)$$

V_0.5_ is the half‐maximal activation voltage and *k* is the slope factor describing the I_hERG_ activation relation.

### Molecular modelling

*Computational docking:* Computational docking of disopyramide enantiomers to hERG models was performed using GOLD (GOLD version 5.6; Cambridge Crystallographic Data Centre, Cambridge, UK) and Flexidock (Flexidock module of SYBYL version 2.0, Certara, Princeton, NJ, USA) as previously described [44, 45]. Template hERG pore structures used were the cryoEM structure of Wang and MacKinnon [6] and a well-characterized homology model of the hERG pore built on the structure of the MthK K^+^ channel [44, 45]. The shape of the drug binding cavity below the selectivity filter differs somewhat between MthK model and cryoEM structures but the main difference is the disposition of the F656 side chain, which projects towards the K^+^ permeation pathway in the MthK model, allowing interactions with disopyramide that were not found with the cryoEM structures. A comparison of the MthK model with a recent cryoEM structure of hERG is shown in Supplementary Fig. 5. Neither GOLD nor Flexidock resulted in docking outputs consistent with enantiomer selectivity, measured either as energy scores or correspondence with the experimental data presented here, and details of docking are therefore only described in the Supplement (Supplementary Fig. 6). For both enantiomers the MthK-based hERG pore model produced lower energy score docking outputs compared with the cryoEM structural model and these docking outputs were used for separate molecular dynamics (MD) simulations of (S(+) and R(−) disopyramide bound in the hERG pore that was embedded within hydrated phospholipid bilayer patches.

*Molecular dynamics simulation:* A homology model of the hERG pore built on the template structure of the open pore MthK structure and including the S5 helix (see [45] for model alignment) was incorporated into a POPC bilayer patch containing 256 lipid molecules in each membrane layer, using Gromacs tools. The model was hydrated with a 15 angstrom layer of water (above and below the membrane) containing K^+^ and Cl^−^ ions equivalent to a KCl concentration of 150 mM. Simulations were run with the POPC-embedded hERG model containing either R(−) or S(+) disopyramide located in the pore according to the lowest energy score output of the docking of the drugs to the MthK-based hERG homology model (Supplementary Figs. 6 and 7). Partial charges and parameterization of the disopyramide enantiomers were calculated using ACPYPE [46]. The drug-protein-bilayer-water systems were subjected to a 10 ns MD run at 310 K using Gromacs [47], with protein and drug heavy atoms constrained to allow lipids to “anneal” against the protein surface and solvent waters to equilibrate and solvate the pore. The atom constraints were then removed for 200 ns of unconstrained MD at 310 K. Starting structures for the simulations are included in the Supplementary information.

## Results

### ***Concentration-dependent I***_***hERG***_*** inhibition by disopyramide enantiomers***

**Fig. 1:**
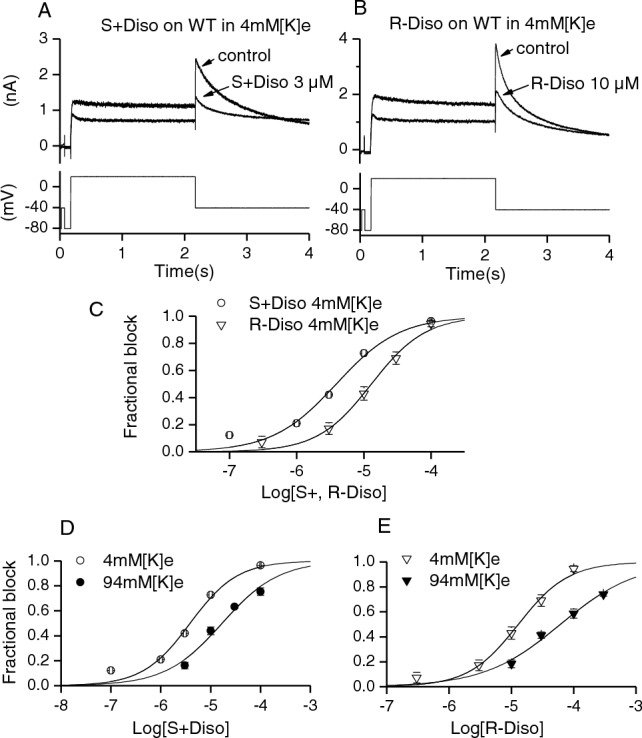
Effects of S(+) and R(−) Disopyramide on WT I_hERG_. Representative I_hERG_ traces before (control) and after achieving steady-state block by S(+) disopyramide (**A**) and R(−) disopyramide (**B**) with the voltage protocol shown underneath. **C** shows concentration-response relation for S(+) and R(−) disopyramide (n = 5–7 for S(+) and n = 4–6 for R(−) disopyramide). **D, E** show rightwards shift of the concentration–response relation for S(+) disopyramide (**D**) and R(−) disopyramide (**E**) in 94 mM[K^+^]_e_ Tyrode’s solution(n = 5–7 for both S(+) and R(−) disopyramide) compared with 4 mM [K^+^]_e_ Tyrode’s

The voltage protocol for determining the sensitivity of I_hERG_ to S(+) and R(−) disopyramide was comprised of a 2 s depolarizing voltage command to + 20 mV from a holding potential of − 80 mV, followed by a 4 s repolarizing step to – 40 mV (protocol applied every 12 s, e.g. [27, 39]). The protocol was applied continuously in control and during exposure to disopyramide, with drug effects typically reaching a steady-state within 3 min of application. Figure 1A shows representative traces in the absence (control) and presence of S(+) disopyramide (S(+) diso at 3 μM; Fig. 1A) or R(−) disopyramide (R(−) diso at 10 μM Fig. 1B). Five concentrations of each enantiomer were tested and for each concentration the mean fractional block of the I_hERG_ tail at – 40 mV was calculated (Eq. 1) and plotted as shown in Fig. 1C. The half maximal inhibitory concentration (IC_50_) for WT I_hERG_ inhibition derived from a fit to the data with Eq. 2 was 3.9 µM (± 0.6 µM) for S(+) disopyramide (Hill coefficient (*h*) of 0.9 ± 0.2), whilst it was 12.9 µM (± 1.4 µM) for R(−) disopyramide (*h* of 1.1 ± 0.1). This compares with an IC_50_ for WT I_hERG_ by racemic disopyramide obtained under identical recording condition of 7.3 µM (*h* of 0.9 ± 0.08) [27], a value that is intermediate between those found here for the two enantiomers. Figure 1D and E show concentration–response relations for inhibition of outward I_hERG_ tails (as shown in Fig. 1C) and for inward K^+^ flux, produced by measuring inward I_hERG_ tails at − 120 mV with raised (94 mM) [K^+^]_e_ (cf [27]) (Supplementary Fig. 1). The potency of I_hERG_ block was lower for inward I_hERG_ in high [K^+^]_e_, for both the S(+) and R(−) enantiomers (with IC_50_ values respectively 4.3 and 4.7-fold those for outward I_hERG_ tails; see Table 1). This has been previously observed for the racemate [27] and is consistent with a competition between K^+^ ions and drug for an internal K^+^ binding site within the channel pore.

**Table 1:** list of mutants tested with its IC_50_*, h v*alue, tested concentration range and fold change

S + DISO	IC_50_(µM)	*h *value	(µM) *n* for each	Fold of WT
WT	3.9 ± 0.6	0.9 ± 0.2	0.1–100 (*n* = 5–7)	
WT (inward 94 mM [K^+^])	16.9 ± 3.0	0.8 ± 0.1	3–100 (*n* = 5–7)	4.3
N588K	3.4 ± 0.5	0.8 ± 0.1	0.1–100 (*n* = 4–7)	0.9
N588E	3.3 ± 0.2	0.5 ± 0.02	0.1–100 (*n* = 5–8)	0.8
Y652A	189.9 ± 33.7	1.0 ± 0.2	30–1000 (*n* = 5)	48.7
D-Y652A	65.1 ± 21.8	0.54 ± 0.1	1–1000 (*n* = 5–11)	16.7
F656A (inward 94 mM [K^+^])	497.8 ± 16.8	0.9 ± 0.03	30–1000 (*n* = 5–9)	29.5
S631A	4.8 ± 0.4	0.7 ± 0.04	1–300 (*n* = 5–6)	1.2

R- DISO	IC_50_(µM)	*h *value	(µM) *n* for each	Fold of WT
WT normal	12.9 ± 1.4	1.1 ± 0.1	0.3–100 (*n* = 4–6)	3.3 (of S +)
WT (inward 94 mM [K^+^])	61.1 ± 7.4	0.7 ± 0.1	10–300 (*n* = 5–7)	4.7(3.6 of S +)
N588K	51.2 ± 4.7	0.9 ± 0.1	3–1000 (*n* = 4–8)	4.0 (0.8 of S +)
N588E	9.1 ± 1.1	0.69 ± 0.05	0.3–100 (*n* = 4–8)	0.7(2.8 of S +)
Y652A	145.5 ± 18.3	1.0 ± 0.1	30–1000 (*n* = 5–7)	11.3(1.0 of S +)
D-Y652A	67.7 ± 8.9	0.7 ± 0.1	3–1000 (*n* = 5–7)	5.2(1 of S +)
F656A (inward 94 mM [K^+^])	1200.0 ± 201	0.8 ± 0.1	30–5000 (*n* = 5–8)	19.6(2.4 of S +)
S631A	31.4 ± 7.5	0.9 ± 0.2	3–300 (*n* = 5–6)	2.4(6.5 of S +)
N588K-Y-up	4.3 ± 0.4	0.6 ± 0.03	0.1–1000 (*n* = 5–8)	
N588K-Y-down	63.4 ± 2.3	0.6 ± 0.1	1–3000 (*n* = 5–6)	
N588K-F-up	151.7 ± 59.3	0.7 ± 0.2	1–3000 (*n* = 5)	

We previously reported that application of disopyramide racemate causes a leftward shift in voltage-dependent WT I_hERG_ activation [27]. To investigate the role of both enantiomers in the modulation of WT I_hERG_ activation, we tested the voltage dependence of inhibition by using a 2 s voltage-command to a range of test potentials and monitoring outward I_hERG_ tails at − 40 mV [27, 48]. Current–voltage (I–V) relations were evaluated for WT I_hERG_ in the absence and presence of S(+) and R(−) disopyramide. From individual cell data, I_hERG_ activation parameters were obtained and plotted as shown in Supplementary Figs. 2Ai and 2Aii. For WT I_hERG_ the activation V_0.5_ was − 19.9 ± 1.5 mV in control and − 30.8 ± 2.2 mV in presence of 3 µM S(+) disopyramide (n = 7 cells, *p* < 0.01). For modulation of WT I_hERG_ activation by R(−) disopyramide, V_0.5_ was − 12.8 ± 2.7 mV in control and − 21.3 ± 1.6 mV in presence of 10 µM R(−) disopyramide (n = 8 cells, *p* < 0.01.There was no significant change to the slope of the activation relationship (with *k* values of 6.1 ± 0.3 mV and 5.9 ± 0.7 mV in control and in presence of S(+) disopyramide and 6.3 ± 0.3 mV and 5.8 ± 0.8 mV in control and in presence of R(−) disopyramide; *p* > 0.05). The voltage-dependence of I_hERG_ blockade by disopyramide enantiomers was quantified by plotting mean fractional block against test potential, as shown in Supplementary Fig. 2B. For 3 μM S(+) -disopyramide, inhibition increased progressively up to − 10 mV and levelled out between 0 and + 60 mV (Supplementary Fig. 2Bi). I_hERG_ inhibition by 10 μM R(−) disopyramide also exhibited voltage dependence with I_hERG_ inhibition increasing up to 0 mV and then levelling out between 10 and + 60 mV (Supplementary Fig. 2Bii). Thus, both enantiomers exhibited voltage-dependent I_hERG_ inhibition.

### Effects of the N588K and N588E mutations on the potency of action of disopyramide enantiomers

**Fig. 2:**
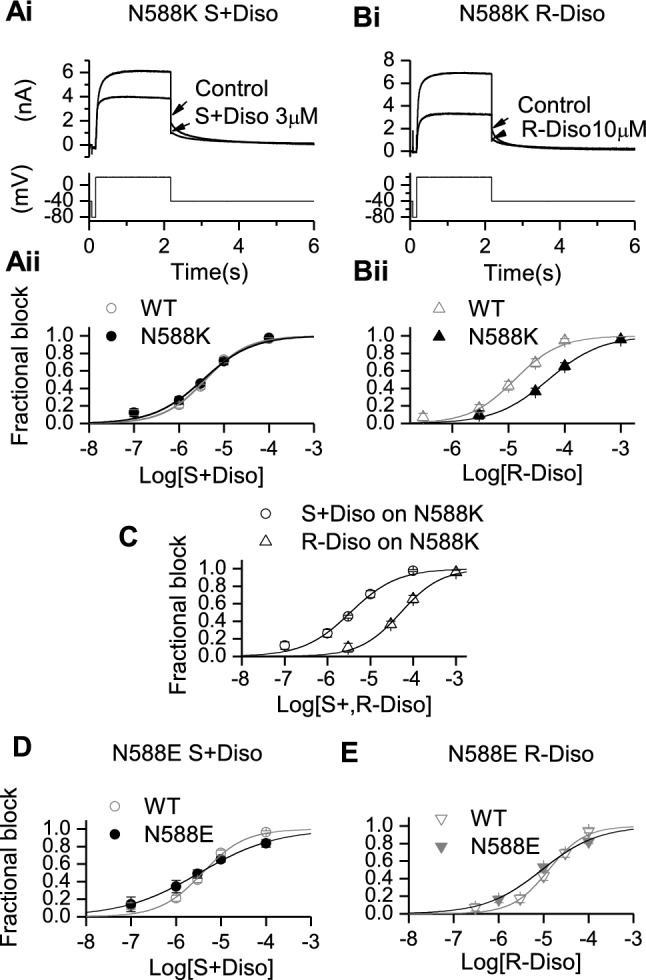
Effects of mutant N588K and N588E on I_hERG_ inhibition by S(+) and R(−) Disopyramide. Representative traces of N588K I_hERG_ in the absence and presence of S(+) disopyramide (**Ai**) and R(−) disopyramide (**Bi**), elicited by the voltage protocol shown in the lower panel. **Aii**, **Bii**. Concentration–response relations for N588K I_hERG_ block compared with that for WT hERG for S(+) disopyramide (**Aii,** n = 4–7 for N588K) and R(−) disopyramide (**Bii,** n = 4–8 for N588K) or compared with each other (**C**). **Di** and **Dii** concentration–response relations for N588E I_hERG_ block compared with WT for S(+) disopyramide (**Di,** n = 5–8 for N588E) and R(−) disopyramide (**Dii,** n = 4–8 for N588E)

The single amino-acid change from Asn (N) to Lys (K) in the N588K mutation involves a switch from a neutral to a positively charged residue in the external S5-Pore linker region of the channel and produces a marked positive shift in voltage-dependent inactivation [36, 49, 50]. Interrogation of N588K I_hERG_ inhibition is valuable as this mutation significantly attenuates inactivation and yet is distant to residues known to contribute to drug binding. This mutation is clinically significant, giving rise to the SQT1 variant of short QT syndrome [49] and exerts only a modest effect (1.5-fold IC_50_ elevation) on I_hERG_ blocking potency of racemic disopyramide [9, 25]. The effect of the two disopyramide enantiomers on N588K I_hERG_ was investigated using the same voltage protocol used to study WT I_hERG._ Figure 2Ai shows representative traces before application (control) and in the presence of S(+) disopyramide (Ai, S(+) DISO 3 μM), whilst Fig. 2Bi shows representative traces for R(−) disopyramide (Bi, R(−) DISO 10 μM). Figures 2Aii and Bii respectively show plotted concentration response data for S(+) and R(−) enantiomers. The derived IC_50_ for N588K I_hERG_ inhibition by S(+) disopyramide was 3.4 µM (± 0.5 µM, *p* > 0.05 versus WT) with *h* of 0.8 ± 0.1 (*p* > 0.05 versus WT). For R(−) disopyramide the derived IC_50_ was 51.2 µM (± 4.7 µM, *p* < 0.0005 versus WT) with *h* of 0.9 ± 0.1 (*p* > 0.05 versus WT), which was 15 fold the S(+) disopyramide IC_50_ elevation. Therefore, disopyramide inhibition of N588K I_hERG_ exhibited pronounced stereoselectivity, as shown by the overlain concentration-response relations in Fig. 2 C for the two enantiomers. Qualitatively similar results were also observed for a second attenuated inactivation mutant, S631A (see Table 1 and Supplementary Fig. 3).

By contrast with N588K, the N588E mutation (Asn to a negatively charged Glu) produces a marked negative shift in voltage dependent inactivation [51] and offers a valuable comparator for the N588K mutation in studying hERG pharmacology [8]. Consequently, the effects of S(+) and R(−) disopyramide on N588E I_hERG_ were evaluated. Due to the gating changes of N588E hERG, its pharmacology is best studied through observing drug effects on inward I_hERG_ tails at a negative voltage (see Supplementary Fig. 4A), following an activating command [8]. Mean concentration response data were plotted as shown in Figs. 2D (S(+) disopyramide) and 2E (R(−) disopyramide) overlain with corresponding WT I_hERG_ data, from which, the generated IC_50_ and *h* values in N588E showed no statistically significance to its corresponding WT (*p* > 0.05 see Table 1), so the potency of I_hERG_ inhibition by S(+) and R(−) disopyramide was comparatively little affected by the N588E mutation, even though there was a small increase in potency for R(+) disopyramide relative to the S(+) disopyramide for the N588E mutation.

### Effects of the F656A and Y652A mutations on the potency of action of disopyramide enantiomers

**Fig. 3:**
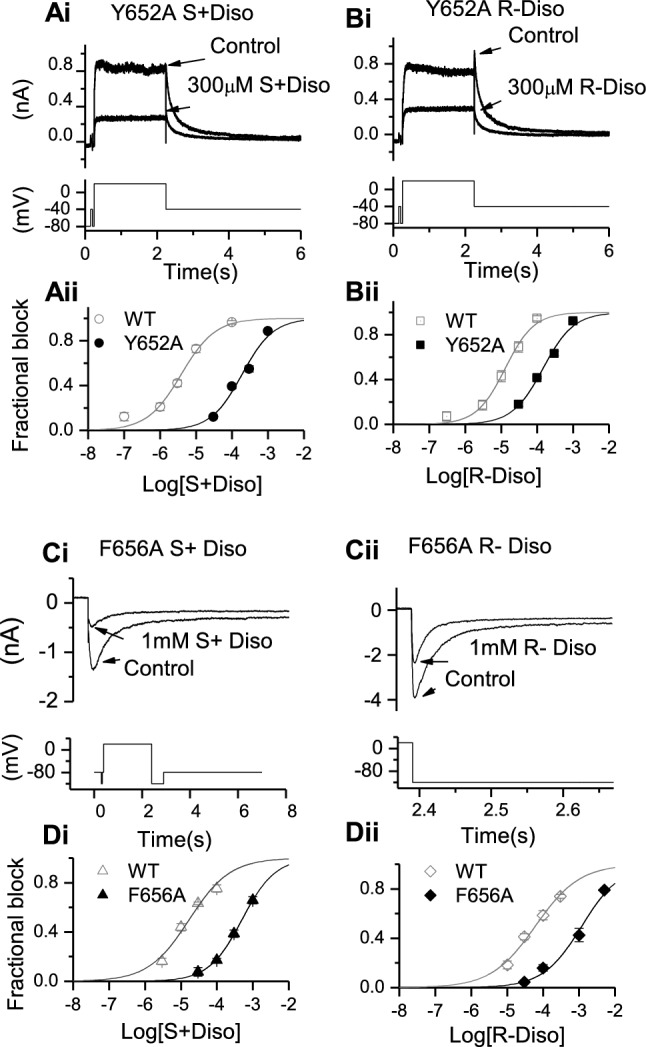
Effects of S6 aromatic mutants Y652A and F656A on S(+) and R(−) disopyramide block of I_hERG_. Representative traces of Y652A I_hERG_ in the absence and presence of S(+) disopyramide (**Ai**) and R(−) disopyramide (**Bi**)**,** elicited by the voltage protocol shown in the lower panel. **Aii, Bii**. Concentration–response relations for Y652A I_hERG_ block compared with WT for S(+) disopyramide (**Aii,** n = 5–11 for Y652A**)** and R(−) disopyramide (**Bii,** n = 5–7 for Y652A). Representative traces of F656A I_hERG_ in the absence and presence of S(+) disopyramide (**Ci**) and R-disopyramide (**Cii**)**,** elicited by the whole voltage protocol (left) or on expanded timescale (right), shown in the lower panels. **Di, Dii.** Concentration–response relations for F656A I_hERG_ block compared with WT for S(+) disopyramide (**Di,** n = 5–9 for F656A**)** and R(−) disopyramide (**Dii,** n = 5–8 for F656A).

The S6 aromatic residues F656 and Y652 have been implicated as key determinants of I_hERG_ inhibition by a range of drugs (e.g. [1, 4, 5, 27, 39, 45]). Importantly, the Y652A and F656A mutations have been shown to be critically important for I_hERG_ inhibition by racemic disopyramide [27]; this is in contrast to residues near the base of the selectivity filter/pore helix (T623A and S624A), mutations to which have been found not to affect disopyramide potency [27]. Therefore, we investigated the effects of the Y652A and F656A mutations on the actions of S(+) and R(−) disopyramide. Figure 3Ai and Bi show representative Y652A I_hERG_ traces in the absence and presence of a high concentration (300 µM) of the two enantiomers, whilst Fig. 3Aii and Bii show concentration–response relations respectively for S(+) and R(−) disopyramide, with those for WT controls shown superimposed. Consistent with prior data for the racemate [27], the inhibitory effect of both enantiomers on I_hERG_ was weakened by the Y652A mutation. However, the impact of this mutation was greater on the potency of S(+) disopyramide than on that of the R(−) enantiomer, with IC_50_ values of 48.7-fold and 11.3-fold those of their respective WT controls (see Table 1).

Figure 3Ci and Cii show representative inward I_hERG_ tails carried by F656A I_hERG_. The full protocol is shown as an inset to Fig. 3Ci, whilst Fig. 3Cii shows alignment of the hyperpolarizing step to −120 mV with the inward tail currents shown. Figure 3Ci shows effects of 1 mM S(+) disopyramide on inward current tails, whilst Fig. 3Cii shows effects of 1 mM R(−) disopyramide. Figures 3Di and Dii show concentration response relations for the two enantiomers on F656A I_hERG_, with WT control data superimposed. The I_hERG_ IC_50_ for S(+) disopyramide was 29.5-fold that for its WT control, whilst that for R(−) disopyramide was 19.6-fold that of its WT control (see Table 1). Thus, I_hERG_ block by both enantiomers was sensitive to mutation of Y652 and F656, with the inhibitory potencies of the S(+) enantiomer affected more by these mutations than those of the R(−) enantiomer.

**Fig. 4:**
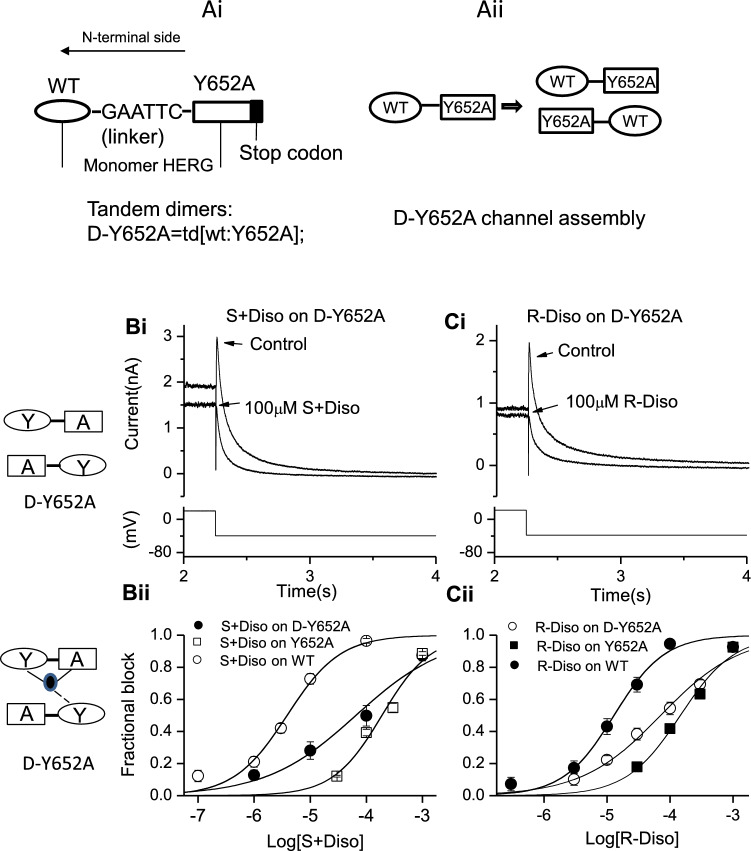
Effects of S6 tandem dimer D-Y652A (td [wt: Y652A]) on S(+) and R(−) disopyramide block of I_hERG_. (**Ai**) Tandem dimers D-Y652A (td[wt:Y652A]) are shown as a concatenated molecule of the monomer WT(N-terminal) and monomer Y652A (C-terminal) with a short linker. (**Aii**) When functional channels incorporating dimers assemble, mutation sites should be located on subunits in the diagonal position of the tetrameric channel, WT subunits are indicated by ovals and the mutated subunits by rectangles. (**Bi**) Upper left inset panel shows the assembly of D-Y652A, oval represents WT monomer with residue Y in position 652, and rectangle represents Y652A mutant monomer with residue A in position 652. Lower left panel represents the possible contribution of residues to the binding on the subunits. Filled oval represent enantiomers interacting with residues on the subunits (solid lines indicate positive interactions, and dashed lines indicate possible interactions). Representative traces of tandem dimer Y652A I_hERG_ in the absence and presence of S(+) disopyramide (**Bi**) and R(−) disopyramide (**Ci**), elicited by the voltage protocol shown in the lower panel. (**Bii**, **Cii**). Concentration–response relations for WT, the tandem dimer Y652A I_hERG_ and homotetrameric Y652A I_hERG_ block by S(+) disopyramide (**Bii**, n = 5–11 for D-Y652A**)** and R-disopyramide (**Cii**, n = 5–7 for D-Y652A)

As the inhibitory potency of S(+) enantiomer inhibition of I_hERG_ appeared particularly strongly affected by the Y652A mutation, further experiments were performed to investigate further the role of this residue, using WT-Y652A tandem dimers (see Fig. 4A and [34, 35]). Figures 4Bi and Bii show concentration response relations for S + disopyramide on WT-Y652A tandem dimer I_hERG_ for each enantiomer plotted together with those for WT and Y652A I_hERG_. Figures 4Ci and Cii show corresponding data for R(−) disopyramide. For both enantiomers the observed potency of inhibition of WT-Y652A tandem dimer I_hERG_ was intermediate between that observed for WT I_hERG_ and that for Y652A I_hERG_ (see Table 1 for IC_50_ values). This indicates that interactions with multiple Y652 residues (and probably Y652 on adjacent subunits) are required for disopyramide to bind optimally to the hERG channel.

### Rescue of inhibitory potency of R- disopyramide on N588K hERG

**Fig. 5:**
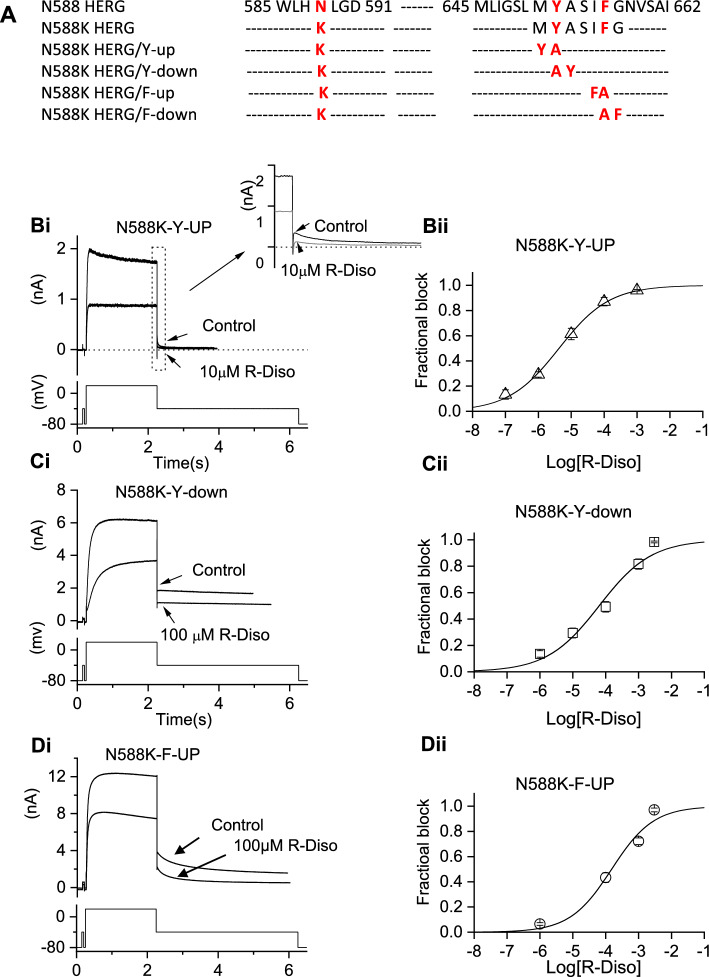
Repositioning of aromatic residues on an N588K background. (**A**) Schematic showing sequences of S5-pore and S6 domain regions to show location of N588K and repositioning N588K-up or down mutants. (**B-D**) effects of repositioning S6 aromatic residue Y652 and F656 in attenuated inactivation N588K channels on I_hERG_ blocking potency of R(−) disopyramide. Representative traces of N588K-Y-up I_hERG_ in the absence and presence of R(-) disopyramide (**Bi,** n = 5–8 for N588K-Y-up**),** elicited by the voltage protocol shown in the lower panel and its concentration-response relation (**Bii**), Representative traces of N588K-Y-down I_hERG_ in the absence and presence of R(−) disopyramide (**Ci,** n = 5–6 for N588K-Y-down), elicited by the voltage protocol shown in the lower panel and its concentration-response relation (**Cii**). Representative traces of N588K-F-up I_hERG_ in the absence and presence of R(−) disopyramide (**Di**, n = 5–8 for N588K-F-up**),** elicited by the voltage protocol shown in the lower panel and its concentration–response plot (**Dii**)

Figure 2 provides evidence that a marked difference between S(+) and R(−) disopyramide on N588K was a differential requirement for inactivation competence for I_hERG_ inhibition to occur. Given that the N588 residue lies outside the pore cavity in which S6 helical residues important for disopyramide inhibition reside, it seems likely that the role of inactivation is to influence positioning of binding residue(s) [37], which in turn differentially affects interactions with the two disopyramide enantiomers. To test this proposition, experiments were performed in which the Y652 and F656 residues in the N588K channel were repositioned by one residue, either higher (in the N terminal direction: Y-up; F-up) or lower (in the C terminal direction: Y-down; F-down) in the pore cavity (see Fig. 5A and [37]). Figure 5Bi shows representative records for N588K-Y-up I_hERG_ in the absence and presence of 10 µM disopyramide (with the inset to Fig. 5Bi showing expanded records of tail currents). Figure 5Bii shows the concentration response relation for inhibition of N588K-Y-up I_hERG_. The observed IC_50_ for R(−) disopyramide inhibition of this mutation was 4.3 ± 0.4 µM (*h* of 0.6 ± 0.03), which is substantially lower than that for N588K alone (51.2 ± 4.7 µM; *p* < 0.0001; Fig. 2 and Table 1) and for that for R(−) disopyramide on WT I_hERG_ (Fig. 1 and Table 1) and closer to that for S(+) disopyramide on the WT channel (see Table 1). In contrast, when N588K-Y-down was tested (Fig. 5Ci and Cii), the observed IC_50_ was 63.4 ± 2.3 µM (*h* of 0.6 ± 0.1), which is broadly comparable to that seen for N588K alone (Table 1). Similar experiments were also performed in which the Phe residue at F656 was repositioned. We were unable to record I_hERG_ from cells transfected with N588K-F-Down. Figure 5 Di and Dii show representative records for N588K-F-up. In contrast to N588K-Y-up, this repositioning of the F residue did not result in increased potency of inhibition relative to N588K alone (Table 1), as the IC_50_ for N588K-F-up was 151.7 ± 59.3 µM (Fig. 5Dii, Table 1). Thus, N588K-Y-up but not N588K-F-up rescued I_hERG_ potency of R(−) disopyramide compared to N588K alone.

### Computational modeling of disopyramide enantiomer interactions with hERG

Previous data from mutagenesis and electrophysiology experiments using racemic disopyramide, revealed that the drug binds to the hERG channel within the inner cavity, without a requirement for direct interactions with residues at the base of the channel’s pore helix [27]. Consistent with mutagenesis data, docking simulations conducted using a MthK-based open pore homology model showed low energy binding configurations in which the drug was located within a cluster of aromatic side chains provided by Y652 and F656 [27]. Although cryo-EM structures of hERG have subsequently been obtained [6, 52, 74], the side-chains of F656 lie within hydrophobic “pockets” in these structures and are oriented away from the pore cavity; docking to the cryoEM structure has been shown to provide results for some drugs that are difficult to reconcile with mutagenesis data [45, 53, 54]. Here, docking of both disopyramide enantiomers within the hERG cryoEM structure resulted in location of the drug within the pore cavity below the selectivity filter even for docking runs in which the drug was biased to bind in or near a hydrophobic pocket, and there were only minimal interactions of disopyramide enantiomers with the F656 side chain (not shown) in contradiction with experimental observations of a significant role for F656 in drug binding ([27] and Table 1). For this reason docking runs were also made using the MthK-based homology model previously shown to provide a good correspondence between modelled drug interactions with hERG pore side chains and experimental data on likely drug binding residues [34, 35, 44]. Docking of disopyramide enantiomers in this model resulted in improved docking scores relative to those found in the hERG cryoEM structure, and configurations with substantial drug interactions with both Y652 and F656 side chains consistent with experiment (Supplementary Fig. 6). However we were unable to distinguish preferential interactions that might account for the experimentally observed enantiomer selectivity of S(+) disopyramide compared with R(−) disopyramide.

**Fig. 6:**
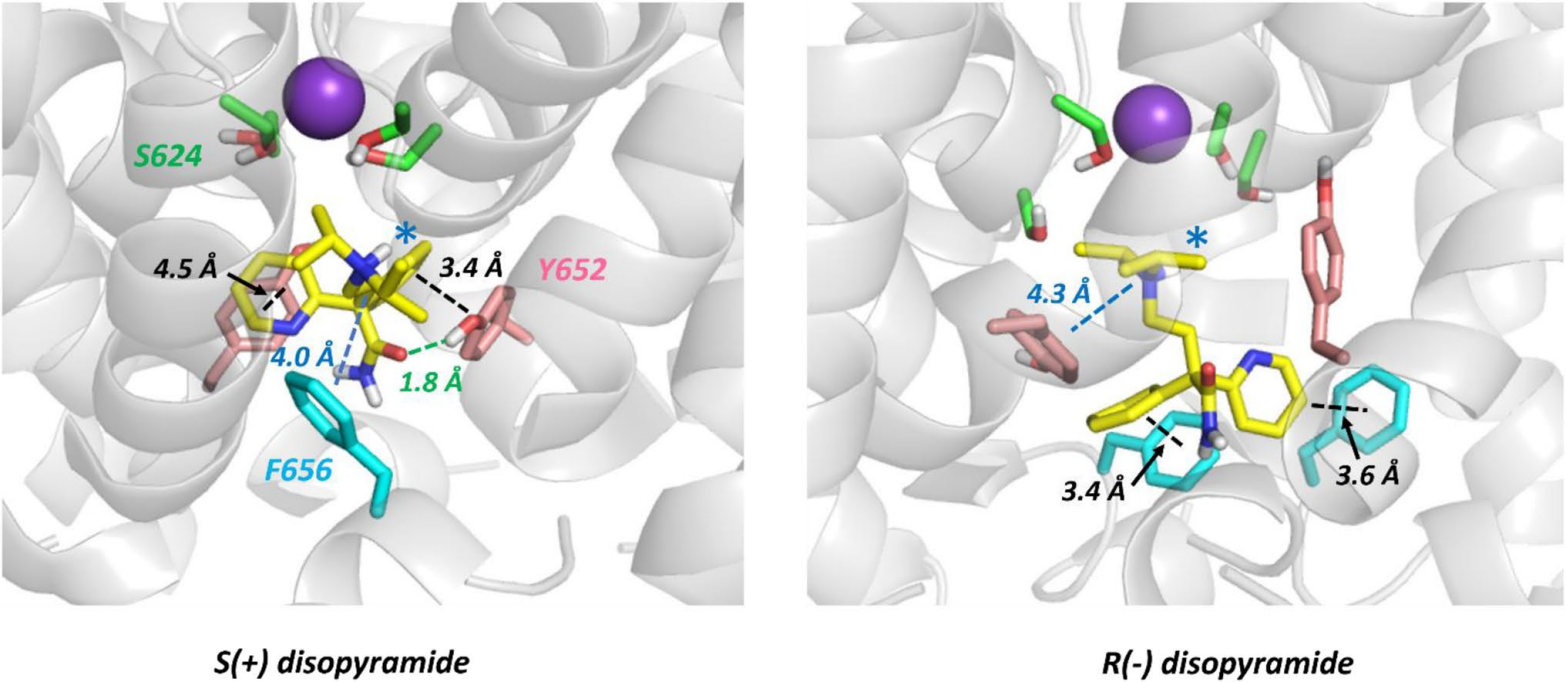
Stereoselective interactions of disopyramide enantiomers within a hERG pore homology model. Each panel is a snapshot of the interaction of a disopyramide enantiomer obtained during 200 ns MD runs within a fully hydrated, membrane-incorporated MthK-based hERG pore homology model (see Supplementary Figs. 6 and 7 for the structure of the membrane system and starting structures of each disopyramide enantiomer within the pore cavity). Disopyramide enantiomers are represented as yellow sticks and hERG pore amino acid side chains are Y652 (pink), F656 (blue) and S624 (green). Discrete drug-pore interactions as defined in [44] are shown as black (aromatic stacking), blue (cation-pi) and green (hydrogen bond) dotted lines. The location of the tertiary aliphatic amino group in or near the binding site for a hydrated K^+^ ion below the selectivity filter is indicated with a blue star. The purple sphere is a K^+^ ion in the 4-position of the selectivity filter. The Y652 side chains that interact with disopyramide lie on adjacent pore subunits in both S(+) and (R−) bound structures. Stereo views of these binding modes are shown in Supplementary Figs. 8 and 9

To explore this further, MD simulations of the disopyramide enantiomers bound within the pore domain of the MthK-based hERG homology model were run in hydrated phospholipid membranes. In these simulations both S(+) and R(−) disopyramide attained pore configurations in which the bulky positively-charged tertiary amino group was located near the internal binding site for a hydrated K^+^ ion (blue stars in Fig. 6) consistent with the reduced binding affinity of both enantiomers by competition with K^+^ ions when IC_50_s were measured with inward K^+^ flow (Table 1). While both enantiomers made stacking interactions with both Y652 and F656 aromatic side chains, S(+) disopyramide made an additional hydrogen bond involving the amide carbonyl oxygen and the aromatic hydroxyl group of a Y652 side chain (green dotted line in Fig. 6). This has the effect of maintaining more substantial interactions with Y652 side chains in the S(+) diso enantiomer run. In the case of the R(−) enantiomer the dominant interactions were with F656 side chains (Fig. 6). In each simulation the disopyramide enantiomer made interactions with two Y652 side chains on adjacent subunits.

## Discussion

### Implications of stereoselectivity of hERG inhibition by disopyramide

Although I_hERG_ block by numerous drugs has been reported [1, 3, 54], relatively few studies have focused on stereoselectivity of I_hERG_ inhibition. The amide local anesthetic drug bupivacaine is a racemate of S(+) bupivacaine (levobupivacaine) and R(−) bupivacaine (dextrobupivacaine): levobupivacaine has been reported to be (∼twofold) more potent at producing I_hERG_ inhibition than dextrobupivacaine [30], with potency of the racemate in-between those of the two enantiomers [31]. The µ-opioid receptor agonist methadone is also chiral: S(+) methadone inhibits I_hERG_ hERG more potently than does R(−) methadone [33], whilst therapeutic activity mainly resides in the R(−) enantiomer. Quinidine and its dextrorotatory diastereomer quinine also exhibit different inhibitory potencies against I_hERG,_ [55, 56]. Quinidine is a more potent I_hERG_ inhibitor than is quinine, though mutation of F656 (F656C) appears to reverse the relative hERG-blocking potency of the two diastereomers [56]. The fact that, in human subjects, infusion of S(+) but not R(−) disopyramide has been reported to result in prolongation of the rate corrected QT interval [29] is indicative of stereoselective effects of the drug on human ventricular repolarization. However, to our knowledge, this is the first study that has demonstrated directly stereoselective inhibition of I_hERG_ by this drug. IC_50_ values for racemic disopyramide inhibition of WT I_hERG_ of ~ 7 µM have been reported [24, 27], while therapeutic total plasma levels reach ~ 6–8 µM [57]. The IC_50_ values of 3.9 and 12.9 µM for S(+) and R(−) disopyramide in the present study lie either side of this range. One comparative study of the pharmacokinetics of S(+) and R(−) disopyramide found mean total plasma concentrations of the two enantiomers to be similar (for similar doses of each enantiomer) at 6.86 μM and 6.33 μM respectively [29], with unbound fractions of 0.208 and 0.338 respectively. We used the hERG IC_50_ values for each of S(+) and R(−) disopyramide to simulate ventricular AP prolongation at respective concentrations of 1.43 μM and 2.14 μM (to mimic unbound concentrations [29]) and observed prolongation of AP duration at 90% repolarization (APD_90_) of 19.3% and 7.4% respectively (see Supplementary Fig. 10). Although simplified (in that these simulations ignore potential enantiomer-selective effects on other ion channels), these results are nevertheless instructive as they are consistent with a greater effect on repolarization of S(+) than R(−) disopyramide in clinical use. To our knowledge, there are no published data on stereoselectivity of fast Na channel current (I_Na_) inhibition by disopyramide. Moreover, as has been noted by others [32], effects of disopyramide enantiomers on depolarization V_max_ (an indirect measure of I_Na_) are controversial: two studies have reported no difference between S(+) and R(−) disopyramide on V_max_ [58, 59], whilst a third study has reported R(−) disopyramide to be more potent [60]. Direct I_Na_ data are therefore clearly needed to determine exactly what the stereoselective effects (if any) of disopyramide are on Na channels. S(+) disopyramide has been reported to produce a smaller negative inotropic effect in humans than does R(−) disopyramide [29]. Bearing in mind the propensity of disopyramide to prolong the QT interval and the associated link to TdP [21, 61], it seems reasonable to link these effects predominantly with the S(+) enantiomer. On the other hand, the reduced sensitivity of I_hERG_ inhibition by S(+) disopyramide to disruption of inactivation seen here, highlights that S(+) disopyramide may be superior to racemic disopyramide for the correction of abbreviated repolarization in SQTS patients with inactivation-attenuating hERG mutations [25, 61, 62]. This benefit may be enhanced by the relative lack of the negative inotropic effect for S(+) disopyramide that is present for the R(−) enantiomer [29]. Conversely, disopyramide is indicated for use in the treatment of obstructive hypertrophic cardiomyopathy (oHCM), in which its negative inotropic action is beneficial as it mitigates hypercontractility [63]. It would therefore be of interest to determine whether R(−) disopyramide might be superior to the racemate and S(+) enantiomer in the treatment of oHCM. From experiments on guinea-pig ileum, both disopyramide enantiomers can exert anti-cholinergic effects, but S(+) disopyramide is 3–4 fold more potent as an anti-cholinergic agent [64]. It is possible, therefore, that when comparing similar concentrations of the two enantiomers, the R(−) enantiomer may be more tolerable from the standpoint of anti-cholinergic side effects than is the S(+) enantiomer. While the Class Ia effects of disopyramide can be explained by its I_Na_ and I_Kr_ blocking effects, there is some evidence that the drug can also inhibit transient outward potassium current, I_TO_ [65–67]. Results regarding the potency of I_TO_ inhibition by disopyramide vary between studies: one study found no effect on rat ventricular I_TO_ at 100 μM [65], while another reported a K_D_ of 259 μM [66]. A further study reported a ~ 33% reduction of rabbit ventricular I_TO_ amplitude by 30 μM [67]. Thus, the drug’s effect on I_TO_ appears to be somewhat less potent than its effect on I_Kr_ and we are unaware of any data on stereoselectivity of the drug’s action on I_TO_.

### ***On the mechanism(s) of stereoselective I***_***hERG***_*** inhibition by disopyramide***

Disopyramide is a gated-state dependent inhibitor of I_hERG_, with little or no binding occurring to closed channels [24, 27]. I_hERG_ block by racemic disopyramide shows voltage-dependence and has been reported to be associated with a leftward shift in I_hERG_ activation [24, 27]. S(+) and R(−) disopyramide share these features of inhibition. Likewise, as reported for the racemate [27], the potency of hERG inhibition for both enantiomers was reduced for inward I_hERG_ in high [K^+^]_e_. This most likely results from a direct interaction (electrostatic repulsion or ‘knock off’) between K^+^ ions and disopyramide positioned in the ion conduction pathway [27, 68, 69]. Disopyramide resides in the pore cavity and, as anticipated [27], I_hERG_ inhibition by both enantiomers was observed to be sensitive to mutations at Y652 and F656; however, the action of S(+) disopyramide was reduced to a greater extent by Y652A and F656A compared to that of R(−) (see fold- changes in Table 1). This indicates that the greater I_hERG_ inhibitory potency of S( +) than R(−) disopyramide is likely to be accounted for by stronger interactions with these key aromatic residues. Additionally, whilst the fold-reduction of I_hERG_ inhibition was markedly greater for Y652A than F656A for S(+) disopyramide, this was not the case for R(−) disopyramide, suggesting different relative roles of the two residues in the binding of each enantiomer. Stereoselectivity of I_hERG_ inhibition by disopyramide was maintained for the F656A mutation but was lost for Y652A, suggesting that Y652A is particularly important for stereoselectivity of disopyramide action. Prior work on block of I_hERG_ by bupivacaine showed stereoselectivity to be abolished by Y652A and reversed by F656A [31], with changes of side chain volume in the mutant channels leading to reshaping of the bupivacaine binding site [31]. Given the importance of the Y652 highlighted in our experiments, we further probed this residue by reducing the symmetry of the tetrameric hERG channel from 4- to 2-fold [34, 35]. Prior work using this approach focused on a subset of high affinity inhibitors (cisapride, terfenadine, E-4031 [34, 35]). Using this approach, if a molecule interacts with a given residue from one subunit, or at most from two diagonally located subunits, then the concentration-response relation for the tandem dimer should lie close to that for the WT channel; on the other hand if the same residue from adjacent subunits contribute to the binding, concentration-dependence for the tandem dimer channel should be located between those of homotetrameric WT and mutant channels [34, 35]. For both S(+) and R(−) disopyramide, the observed potency of inhibition of tandem dimer WT-Y652A (D-Y652A) I_hERG_ was intermediate between that observed for WT I_hERG_ and that for Y652A I_hERG_. This constitutes evidence that each enantiomer interacts with at least two residues from adjacent subunits at position 652 in tetrameric channels to bind optimally.

The low energy configurations of disopyramide enantiomers within the hERG pore model in MD simulations identify interactions of drug, especially the S( +) enantiomer, with Y652 side chains on adjacent (rather than opposing) channel subunits (Fig. 6 and Supplementary Fig. 8) consistent with the tandem dimer experiments. For both enantiomers the location of the positively-charged tertiary aliphatic amino group in or near the internal K^+^ binding site (Fig. 6 and Supplementary Figs. 8 and 9) supports an explanation for reduced drug block by inward K^+^ currents (Table 1) involving competition between drug and K^+^ ions. The MD simulations are also consistent with enhanced interactions of S(+) disopyramide with Y652 chains compared with the R(−) enantiomer (Table 1) and provide a possible explanation for the small enantiomer selectivity of S(+) over R(-) disopyramide; in our simulations the S(+) enantiomer makes an additional hydrogen bond with a Y652 side chain phenolic hydroxyl group that enhances interactions of S(+) with Y652 residues within the pore cavity (Fig. 6 and Supplementary Fig. 8).

A striking feature of our experimental data is the difference in the effect of attenuated inactivation on I_hERG_ inhibition by disopyramide enantiomers. Intact inactivation is crucial for high potency I_hERG_ inhibition of a number of drugs, though for some drugs – including racemic disopyramide – intact inactivation appears somewhat less important [8, 9, 70]. Some evidence suggests that the role of inactivation in drug block of hERG is indirect, potentially mediated via allosteric changes that influence the position of key binding residues (37, 71). For example, in a study of concatenated hERG tetramers, inclusion of a single S620T-containing subunit was sufficient to fully disrupt inactivation, whereas drug potency correlated with the number of S620T subunits in the tetramer [71]. In the same study, the potency of dofetilide but not cisapride was graded according to the number of S631A subunits in concatamers containing that mutation [71]. R(−) disopyramide was more sensitive to both the N588K and S631A mutations (Table 1; Fig. 2B and Supplementary Fig. 3). We selected the N588K mutation for use in the present study because the location of this residue in the external S5-pore linker [49] is remote from the inner cavity binding site for disopyramide [27]. The striking difference between S(+) and R(−) disopyramide in the effects of the N588K mutation suggests that the former is able to adopt favourable binding poses irrespective of the presence or lack of an intact inactivation process. By contrast, R(−) disopyramide was highly sensitive to inactivation attenuation. We reasoned that this was likely to have resulted from a reduced ability of this enantiomer to form favourable interaction with S6 aromatic side chains in the absence of intact inactivation. Repositioning one position up the S6 helix of the tyrosine but not phenylalanine residue against an N588K background markedly increased the potency of R(−) disopyramide towards that for S(+) disopyramide on WT I_hERG_, without restoring WT channel gating (see traces in Fig. 5Bi). This observation supports (i) the key role of the Y652 residue in disopyramide binding and (ii) the notion that N588K produced conformational changes in S6 that reduced the ability of R(−) disopyramide to make favourable binding interactions. These findings are consistent with an earlier observation in which repositioning of aromatic residues equivalent to Y652 and F656 in non-inactivating *eag* channels induced sensitivity to block by cisapride [37] and recent more direct evidence that rotational rearrangements of the S6 helix modulate the configuration of amino acid side chains associated with slow inactivation in Shaker K^+^ channels [72] and with activator binding in KCNQ2 channels [73], respectively.

## Conclusion

The results of this study demonstrate that the S(+) enantiomer of disopyramide is a more potent inhibitor of I_hERG_ than is the R(−) enantiomer. This greater potency is linked to stronger interactions with S6 aromatic binding residues for the S(+) enantiomer: stereoselectivity was lost for Y652A I_hERG_. R(−) disopyramide inhibition was more dependent on intact I_hERG_ inactivation, most likely because of S6 residue orientation changes linked to loss of inactivation. Stereoselectivity of I_hERG_ block by disopyramide occurs at clinically relevant concentrations, with both potentially deleterious and beneficial effects on repolarization (depending on clinical setting) likely to reside predominantly in the S(+) enantiomer. Further studies of stereoselective effects of disopyramide are warranted to identify settings in which preferential use of one or other enantiomer may confer advantages over use of the racemate.

## Supplementary Information

Below is the link to the electronic supplementary material.

Supplementary file1 (DOCX 2306 kb)

## Data Availability

The data for the study are included in the manuscript and Supplementary information. Materials for the study will be made available on reasonable request.

## Code availability

The MthK-based hERG homology model used in this study is available from CED on request.
