# Supplementary material for: Stereoselective block of the hERG potassium channel by the Class Ia antiarrhythmic drug disopyramide
Source: Cell Mol Life Sci. 2024 Nov 28;81(1):466. doi: 10.1007/s00018-024-05498-4 (PMC11604869; doi:10.1007/s00018-024-05498-4)
Supplement: Supplementary file 1 — Supplementary file1 (DOCX 2306 kb) [file 18_2024_5498_MOESM1_ESM.docx]

**ONLINE SUPPLEMENT TO**

**Stereoselective block of the hERG potassium channel by the Class Ia antiarrhythmic drug disopyramide.**

by

**Yihong Zhang^1^, Aziza El Harchi^1^, Andrew F. James^1^, Shigetoshi Oiki^2^, Christopher E Dempsey^3^, Jules C. Hancox^1*^**

**^1^** School of Physiology, Pharmacology and Neuroscience,

University of Bristol,

Biomedical Sciences Building,

University Walk,

BS8 1TD,

Bristol, United Kingdom.

^2^ Biomedical Imaging Research Centre,

University of Fukui

23-3 Matsuokashimoaizuki, Eiheiji-cho,

Fukui 910-1193, Japan

^3^ School of Biochemistry,

University of Bristol,

Biomedical Sciences Building,

University Walk,

BS8 1TD,

Bristol, United Kingdom.

* Author for correspondence

**Supplementary figure 1**: Effects of raised (94 mM) [K^+^]_e_ on S(+) and R(-) disopyramide block of inward I_hERG_

Representative traces of WT inward I_hERG_ tails at -120 mV with raised (94 mM) [K^+^]_e_ in the absence and presence of S(+) Disopyramide (Ai) and R(-) Disopyramide (Aii), elicited by the voltage protocol shown in the lower panel. Concentration-response relations for S(+) Disopyramide (Bi, IC_50_=16.9±3.0 μM, *h*=0.8±0.1, n=5-7) and R(-) Disopyramide (Bii, IC_50_=61.1±7.4μM, *h*=0.7±0.1, n=5-7, see table 1).

*

*

***

***

***

***

*

**Supplementary figure 2:** Effect of S(+) (Ai) and R(-) (Aii) disopyramide on activation parameters. In each case a concentration close to the observed IC_50_ was used (3 µM S(+) disopyramide, n=7; 10 µM R(-)disopyramide, n=8). Mean activation curves were obtained by pooling data from fits to *I*–*V* relations for I_hERG_ tails with a Boltzmann equation to obtain *V*_0.5_ and *k* values(* denotes statistically difference from control at *: *p*<0.05; ***: *p*<0.001, two-way RM analysis of variance (ANOVA) followed by Bonferroni post hoc test). (B) Mean level of fractional block of the I_hERG_ tail at each test potential for 3 µM S(+) disopyramide (see Bi, n=7 and 10 µM R(-) disopyramide (Bii), n=8). The S(+) disopyramide data at all voltages passed Kolmogorov-Smirnov (KS) normality testing, but the R(-) disopyramide data at -40 and -30 mV did not pass KS normality testing. For consistency, the data with each enantiomer (Bi and Bii) were tested with both one-way ANOVA and Kruskal-Wallis (K-W; nonparametric) tests. For S(+) disopyramide 1-way ANOVA gave *p*<0.001 and K-W testing gave *p*<0.02; for R(-) disopyramide, 1-way ANOVA and K-W testing both gave p<0.005. Thus, inhibition of I_hERG_ by both enantiomers was voltage dependent.

**Supplementary figure 3**: Effects of mutant S631A on I_hERG_ inhibition by S(+) and R(-) disopyramide.

Representative traces of S631A I_hERG_ in the absence and presence of S(+) Disopyramide (Ai) and R(-) Disopyramide (Aii), elicited by the voltage protocol shown in the lower panel. Concentration-response plots for S631A I_hERG_ block compared with WT by S(+) Disopyramide (Bi, , IC_50_=4.8±0.4 μM, *h*=0.7±0.04, n=5-6) and R-Disopyramide (Bii, IC_50_=31.4±7.5 μM, *h*=0.9±0.2, n=5-6, see table 1).

**Supplementary figure 4:** Effects of mutant N588E on I_hERG_ inhibition by S (+) and R(-) Disopyramide.

Representative traces of N588E I_hERG_ in the absence and presence of S(+) Disopyramide (Ai) and R(-) Disopyramide(Aii), elicited by the voltage protocol shown in the lower panel. Concentration-response plots for N588E I_hERG_ block compared with WT by S(+) Disopyramide (Bi, IC_50_=3.3±0.2 μM, *h*=0.5±0.02, n=5-8) and R-Disopyramide (Bii, , IC_50_=9.1±1.1 μM, *h*=0.69±0.05, n=4-8, see table1).

**
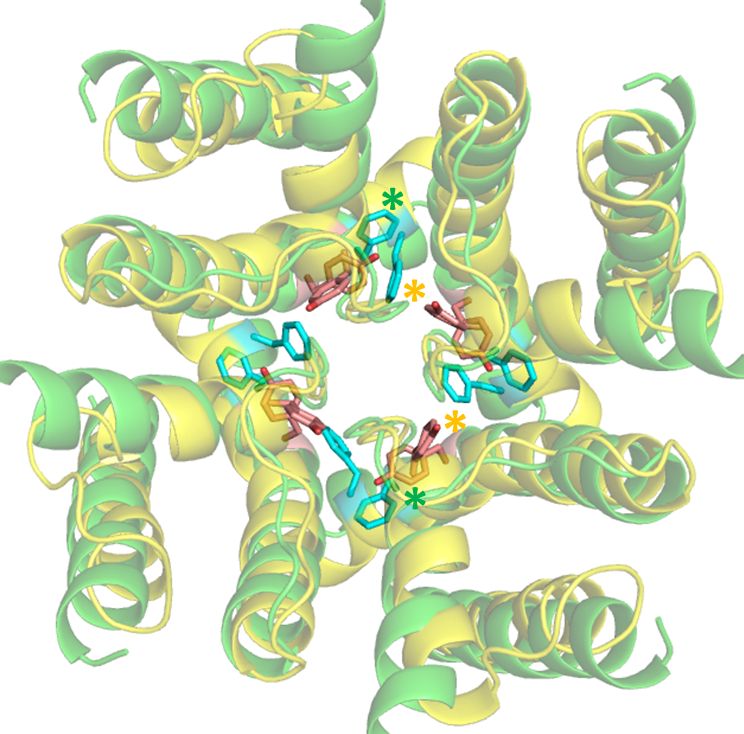
**

**Supplementary figure 5:** Overlay of backbone cartoons of equivalent regions of the MthK hERG homology model (yellow) and a cryoEM structure of hERG in 300 mM KCl in detergent-lipid mixed micelles (PDB: 9CHQ [1]) (green). The side chains of residues Y652 (pink) and F656 (blue) are shown as sticks. The dispositions of Y652 side chains are similar in model and structure (yellow and green stars at bottom of figure) although the side chain rotamers differ. In contrast, the F656 side chains project towards the K^+^ permeation pathway in the MthK model but project away from the K^+^ permeation pathway in hERG cryoEM structures including PDB: 9CHQ shown here (yellow and green stars at top of the figure) and PDB: 5VA2.

**
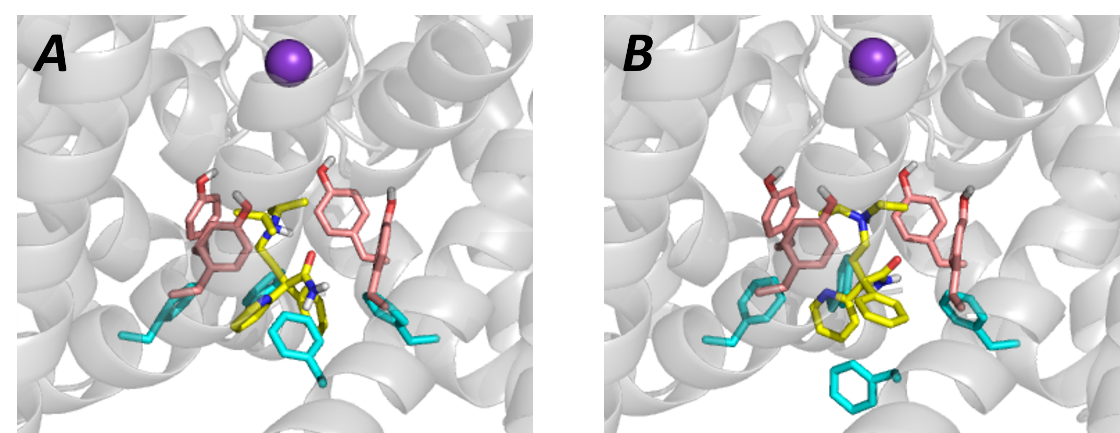
**

**Supplementary figure 6:** Representative low energy score docking outputs for (A) S(+) disopyramide and (B) R(-) disopyramide within the MthK-based hERG pore model obtained using Flexidock. In contrast to docking within the hERG cryoEM structure (PDB: 5VA2), the disopyramide enantiomers can potentially make interactions with multiple F656 sidechains in this model (see text of main paper). These docking outputs were therefore used as starting structures for MD simulations of S(+) and R(-) disopyramide in hydrated POPC membranes to explore enantiomer selectivity.

**
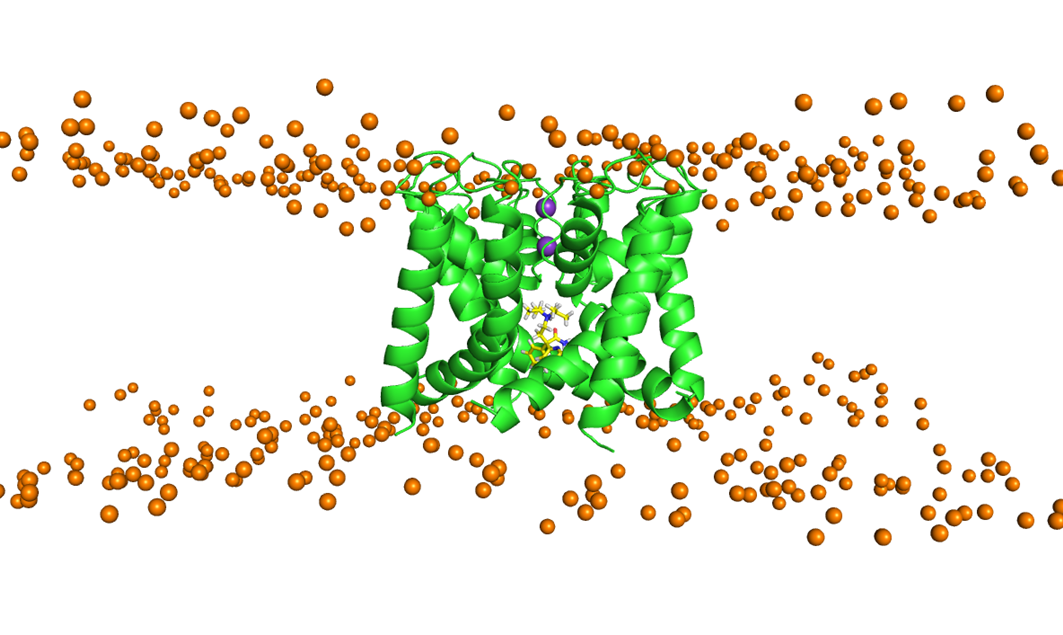
**

**Supplementary figure 7:** Starting structure for 200 ns MD simulation of S(+) disopyramide in the hERG pore model. This structure is the best low energy score output from FlexiDock docking of S(+) disopyramide to the isolated pore model (Supplementary figure 6). In this (and simulations containing R(-) disopyramide and the drug-free pore model simulation) the hERG pore model with or without drug was embedded within a POPC bilayer slab comprising 512 lipids and solvated with a 15 Å layer of water above and below the membrane and MD simulations were run at 310K using Gromacs as described in the main paper. Orange spheres are phosphate phosphorus atoms of POPC lipids. Purple spheres are K^+^ ions in the one and three positions of the selectivity filter.


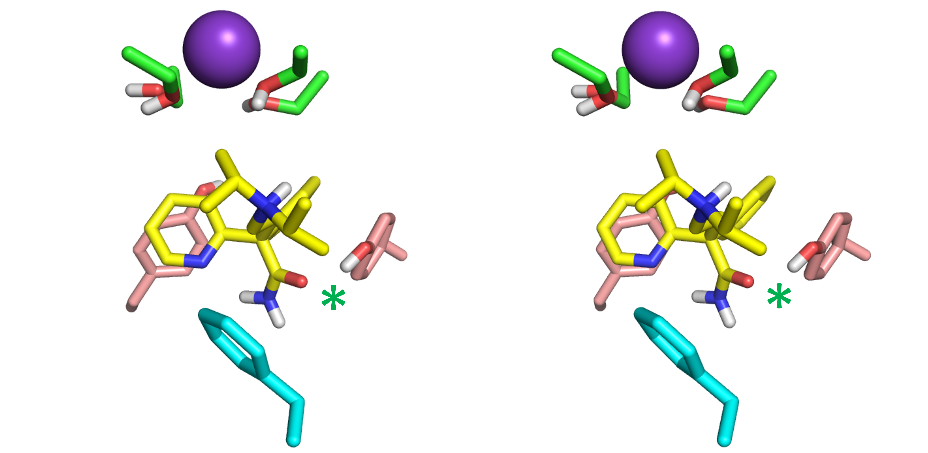


**Supplementary figure 8:** Stereo view of S(+) disopyramide obtained during MD simulation within the MthK-based hERG pore model (this is the same view shown in Figure 6 of the main paper). Y652 (pink) and F656(blue) side chains that make definable interactions with drug are shown. The green star highlights the hydrogen bond between the S(+) disopyramide amide carbonyl and Y652 phenolic hydrogen as described in the main text. The purple sphere is a K^+^ ion in the four-position of the selectivity filter.


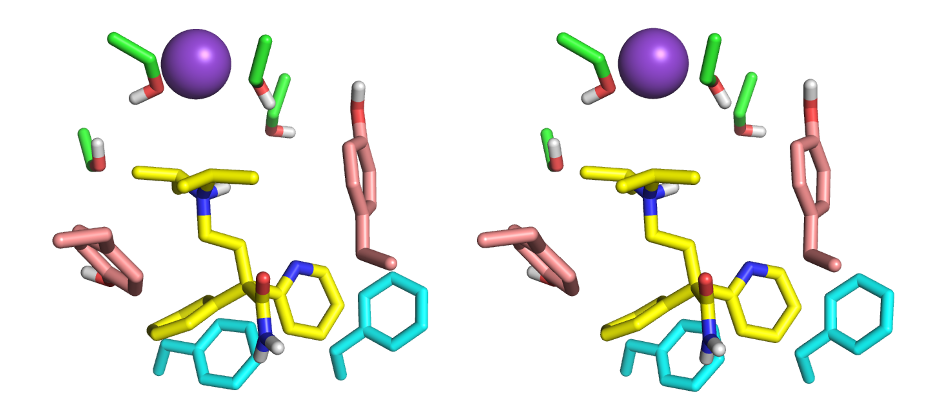


**Supplementary figure 9:** Stereo view of R(-) disopyramide obtained during MD simulation within the MthK-based hERG pore model (this is the same view shown in Figure 6 of the main paper). Y652 (pink) and F656(blue) side chains that make definable interactions with drug are shown. The purple sphere is a K^+^ ion in the four position of the selectivity filter.

**
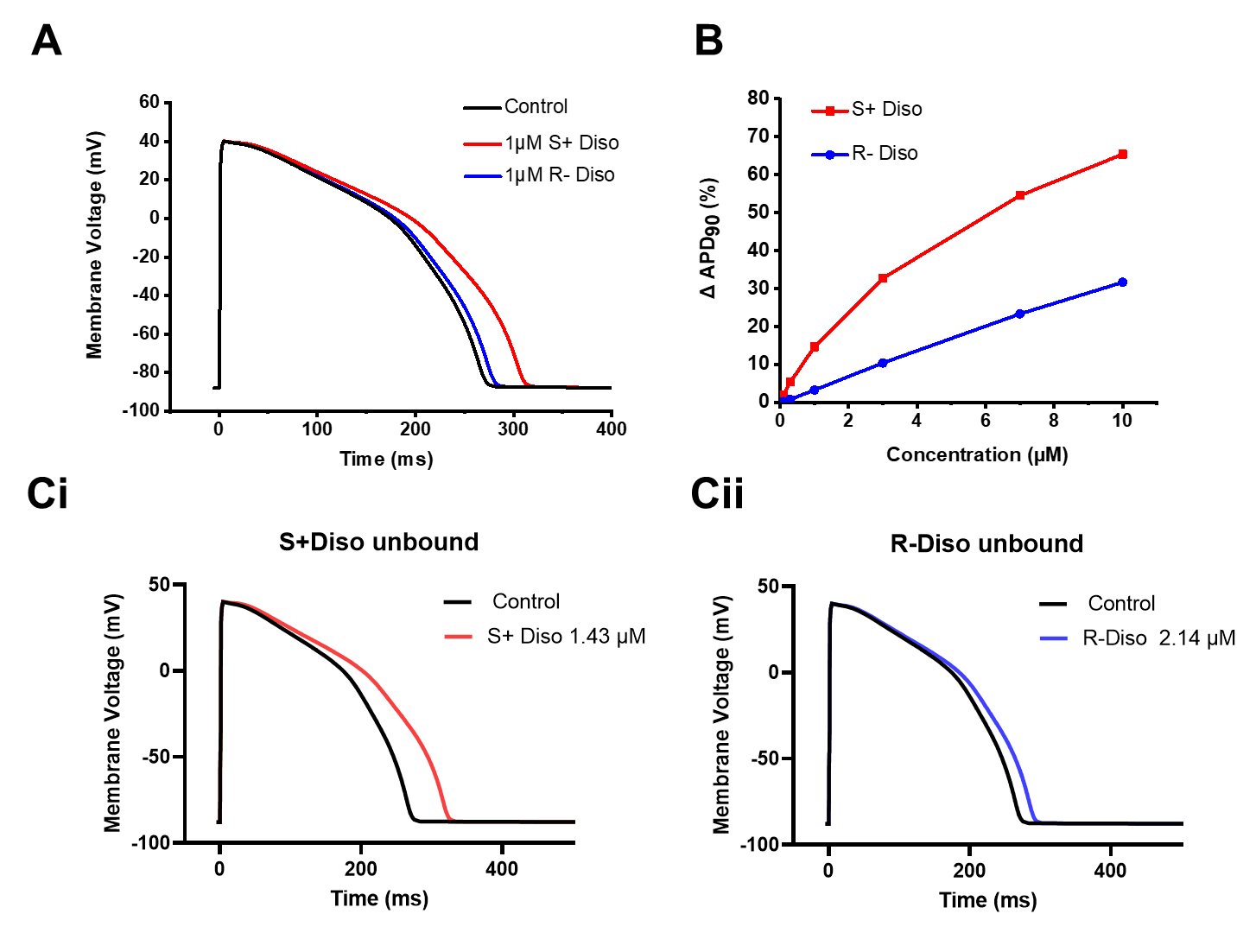
**

**Supplementary figure 10.** Simulated ventricular action potential( AP)prolongation for I_Kr_  block by S(+) and R(-)disopyramide.

(A) shows simulated human ventricular APs in control and with I­_Kr_ block produced by 1μM S(+) and R(-) disopyramide, based on IC_50_ values for WT I_hERG_ block obtained for the two enantiomers. The O’Hara-Rudy CiPA model was run in “[ApPredict](https://www.maths.nottingham.ac.uk/plp/pmzgm/ap_predict/)” web portal software [2]. Stimulation frequency was 1 Hz. (B) shows prolongation of APD_90_ (ΔAPD_90_ (%)) over a range of test concentrations for S(+) and R(+)disopyramide. (C) shows simulated ventricular AP prolongation at concentrations of 1.43 μM S(+) Disopyramide ( Ci) and of 2.14 μΜ R(-) disopyramide(Cii) to mimic unbound concentrations in plasma [3]. Simulations considered I_Kr_ block by disopyramide only and potential effects on other ionic conductances were not included. Further future simulations of enantiomer-selective effects on APD are warranted that will require comparative experimental data of enantiomer effects on other major conductances.

**References**

[1] Lau CHY, Flood E, Hunter MJ, Williams-Noonan, BJ, Corbett, KM, Ng C-A et al. Potassium dependent structural changes in the selectivity filter of hERG potassium channels *Nature Commun,* 2024; 15:7470.

[2] Williams G and Mirams GR A web portal for in-silico action potential predictions*. J Pharmacol Toxicol Methods,* 2015;75:10-16.

[3] Lima JJ, Wenzke SC, Boudoulas H, Schaal SF. Antiarrhythmic activity and unbound concentrations of disopyramide enantiomers in patients. *Ther Drug Monit*, 1990;12:23-28.
